# Dosimetric Comparison of Deep Inspiration Breath-Hold and Free-Breathing Techniques in Stereotactic Body Radiotherapy for Localized Lung Tumors at a Tertiary Care Center

**DOI:** 10.7759/cureus.104466

**Published:** 2026-03-01

**Authors:** Siddharth Arora, Margasagaya Shivkumaran Sathyanarayanan, Suhaildeen Kajamohideen, Srinivas Chilukuri, Kriti Grover

**Affiliations:** 1 Radiation Oncology, Rohilkhand Medical College and Hospital, Bareilly, IND; 2 Surgical Oncology, Sri Ramachandra Institute of Higher Education and Research, Chennai, IND; 3 Pediatric Oncology, Apollo Proton Cancer Centre, Chennai, IND; 4 Pathology, Rohilkhand Medical College and Hospital, Bareilly, IND

**Keywords:** deep inspiration breath-hold, free breathing, lung cancer, motion management, stereotactic body radiotherapy

## Abstract

Background

Respiratory motion is a major source of geometric uncertainty in lung stereotactic body radiotherapy (SBRT) and may lead to increased irradiation of normal lung tissue and adjacent organs at risk (OARs). Deep inspiration breath-hold (DIBH) is an advanced motion management technique that minimizes tumor motion by immobilizing the target during treatment delivery and increasing lung volume. This study aimed to evaluate the dosimetric advantages of DIBH compared with free breathing (FB) in SBRT for patients with localized lung tumors.

Methods

Twenty patients with localized lung tumors underwent computed tomography (CT) simulation using both four-dimensional CT (4DCT) FB and DIBH techniques. SBRT plans were generated for each dataset using 6 MV flattening filter-free (FFF) beams with two coplanar partial arcs (180°-220° offset), and dose calculation was performed using the anisotropic analytical algorithm (AAA) in the Eclipse treatment planning system. Identical dose constraints and plan normalization to the planning target volume (PTV) mean dose were applied for both techniques. Image guidance was performed using cone-beam CT (CBCT). Dosimetric parameters for lung volumes, target coverage, and OAR doses were extracted from dose-volume histograms (DVHs). Statistical comparisons were carried out using paired t-tests.

Results

DIBH significantly increased lung volumes compared with FB, with mean expansion factors of 1.41 and 1.37 for the ipsilateral and contralateral lungs, respectively, and resulted in a 1.45-fold reduction in internal target volume. The mean ipsilateral lung dose was reduced by 36.84% in DIBH plans (7.48 ± 3.57 Gy) compared with FB plans (10.23 ± 4.58 Gy). Statistically significant reductions were also observed in lung V10, V15, and V20 parameters (p < 0.05). DIBH plans demonstrated improved dose conformity (CI: 1.05 ± 0.08) without significant differences in homogeneity or target coverage indices. In addition, doses to critical OARs including the heart, spinal cord, and chest wall were significantly lower in DIBH-SBRT plans.

Conclusion

DIBH-based SBRT provides significant dosimetric advantages over FB SBRT for localized lung tumors by enhancing target immobilization, increasing lung volume, and reducing radiation exposure to the lungs and surrounding critical organs without compromising target coverage. DIBH is a feasible and effective respiratory motion management strategy in lung SBRT, although appropriate patient selection and training are essential for successful implementation.

## Introduction

Lung cancer remains a major global health burden and predominantly affects the elderly population, with a median age at diagnosis of approximately 70 years. It continues to be one of the most frequently diagnosed malignancies and a leading cause of cancer-related mortality worldwide [1]. For patients with early-stage or localized lung tumors who are medically inoperable or unwilling to undergo surgery, stereotactic body radiotherapy (SBRT) has emerged as an established and effective treatment modality [2]. By delivering high radiation doses in a limited number of fractions with steep dose gradients, SBRT achieves excellent local tumor control while limiting exposure to surrounding normal tissues [3].

Despite these advantages, respiratory motion remains a critical challenge in thoracic radiotherapy and is particularly relevant in SBRT due to the high dose per fraction and narrow therapeutic margins [2,3]. Lung tumors undergo continuous displacement during the respiratory cycle, resulting in uncertainties in target localization and dose delivery. Inadequate management of respiratory motion may lead to target underdosage or excessive irradiation of adjacent organs at risk (OARs), including the normal lung, heart, esophagus, and spinal cord. Consequently, achieving reproducible respiratory patterns during both simulation and treatment delivery is essential but remains technically challenging in routine clinical practice [4,5].

To address respiration-related uncertainties, several motion assessment and management strategies have been developed [6,7]. Four-dimensional computed tomography (4DCT) has become integral to modern thoracic radiotherapy planning, enabling visualization and quantification of tumor motion throughout the breathing cycle [8,9]. By correlating respiratory signals, obtained using external markers or airflow-based monitoring systems, with time-resolved CT images, 4DCT generates datasets representing different respiratory phases or amplitudes. This approach reduces motion-induced imaging artifacts compared to conventional 3DCT and facilitates more accurate target delineation [10-12].

Various respiratory motion management techniques are currently employed in SBRT, including motion-encompassing methods, free-breathing (FB) respiratory-gated approaches, breath-hold techniques, and respiratory-synchronized delivery. FB SBRT is widely practiced because of its simplicity and patient convenience; however, it often necessitates larger target margins to compensate for tumor motion, which may increase radiation exposure to surrounding normal tissues [13].

Deep inspiration breath-hold (DIBH) represents an advanced motion management strategy in which radiation is delivered during a controlled breath-hold at deep inspiration. At this phase of respiration, lung volumes are maximized and respiratory-induced tumor motion is substantially reduced, improving geometric stability and increasing the distance between the target and critical organs. In appropriately selected and well-trained patients capable of maintaining reproducible breath-holds of approximately 20 seconds, DIBH offers the potential for improved dose conformity and enhanced sparing of OARs [14].

Given the increasing adoption of SBRT for localized lung tumors and the need to optimize normal tissue protection in high-dose hypofractionated treatments, a direct dosimetric comparison between FB and DIBH techniques is warranted. The present study was designed to perform a paired within-patient comparison of SBRT plans generated using FB and DIBH approaches, with primary emphasis on lung dose-volume parameters and secondary evaluation of target coverage and other OAR dosimetry in patients treated at a tertiary care center.

## Materials and methods

Study design and patient selection

This prospective dosimetric comparative study was conducted at a tertiary care center in Hyderabad, India, over two years from June 2018 to June 2020. The study aimed to compare the dosimetric characteristics of SBRT plans generated using FB and DIBH techniques in patients with localized lung tumors. Eligible patients included those with radiologically confirmed localized lung tumors based on contrast-enhanced thoracic CT. Fluorodeoxyglucose positron emission tomography-computed tomography (FDG PET-CT) was performed, where clinically indicated, to confirm nodal negativity and exclude distant metastasis. Inclusion criteria were as follows: (i) early-stage (T1-T2, N0, M0) non-small cell lung cancer (NSCLC) deemed medically inoperable or refusing surgery; (ii) selected oligometastatic pulmonary lesions (≤3 metastatic lesions confined to the lung); (iii) tumor size ≤ 5 cm; and (iv) Eastern Cooperative Oncology Group (ECOG) performance status 0-2. Patients were required to be able to understand and comply with breath-hold instructions and maintain a reproducible breath-hold of approximately 20 seconds for DIBH eligibility. Exclusion criteria included centrally located ultracentral tumors requiring altered fractionation, severe pulmonary compromise precluding SBRT positioning, inability to cooperate with breath-hold maneuvers, or irregular respiratory patterns.

SBRT rationale and treatment intent

Patients were treated using SBRT according to institutional protocols for medically inoperable early-stage NSCLC and selected oligometastatic pulmonary lesions. SBRT is an established standard of care in this setting due to its ability to deliver ablative radiation doses with high geometric precision [15]. Treatment was delivered with a biologically effective dose (BED) calculated using an α/β ratio of 10 (BED10 ≥ 100 Gy), consistent with evidence demonstrating improved local tumor control rates at this dose threshold [15]. Given the high dose per fraction and steep dose gradients inherent to SBRT, precise target delineation and motion management were considered essential for safe and effective treatment delivery [16]. Respiratory motion was assessed using 4DCT for free-breathing datasets and controlled breath-hold imaging for DIBH plans. The application of advanced imaging and motion management techniques enabled optimization of planning target volume (PTV) margins while maintaining adequate target coverage and adherence to OAR dose constraints [17]. All treatment plans were generated with the intent to achieve high conformity to the target volume, steep dose fall-off, and reproducible delivery under image guidance.

Simulation and imaging protocol

All patients underwent CT simulation in the supine position, with arms elevated above the head using a customized immobilization device to ensure setup reproducibility. Simulation was performed using a dedicated radiotherapy planning CT scanner. Each patient underwent both FB and DIBH CT acquisition during the same simulation session. For FB datasets, 4DCT imaging was performed to capture respiratory-induced tumor motion. Respiratory signals were recorded using an external respiratory monitoring system, and CT images were sorted into phase-based bins representing different stages of the breathing cycle. For DIBH acquisition, patients underwent pre-simulation breath-hold training with visual coaching. CT images were obtained during stable deep inspiration using respiratory monitoring to ensure reproducibility. Multiple breath-hold acquisitions were obtained when necessary to verify consistency before contouring. Image guidance during treatment delivery was performed using gated DIBH cone-beam CT (CBCT).

Target and OAR delineation

Target delineation was performed separately on both FB and DIBH datasets. For the FB technique, the gross tumor volume (GTV) was contoured on all respiratory phases of the 4DCT. An internal target volume (ITV) was generated encompassing the composite tumor motion across phases. A uniform 5 mm isotropic margin was added to the ITV to create the PTV, accounting for setup uncertainties. For DIBH plans, the GTV was delineated on the breath-hold CT dataset. As per the SBRT protocol, the clinical target volume (CTV) was considered equivalent to the GTV. A reduced isotropic margin of 3 mm was added to generate the PTV, reflecting reduced respiratory motion during breath-hold. OARs, including bilateral lungs, heart, esophagus, spinal cord, and chest wall, were contoured independently on both FB and DIBH datasets according to institutional protocols and international SBRT contouring guidelines.

Treatment planning and dosimetric evaluation

**Figure 1:**
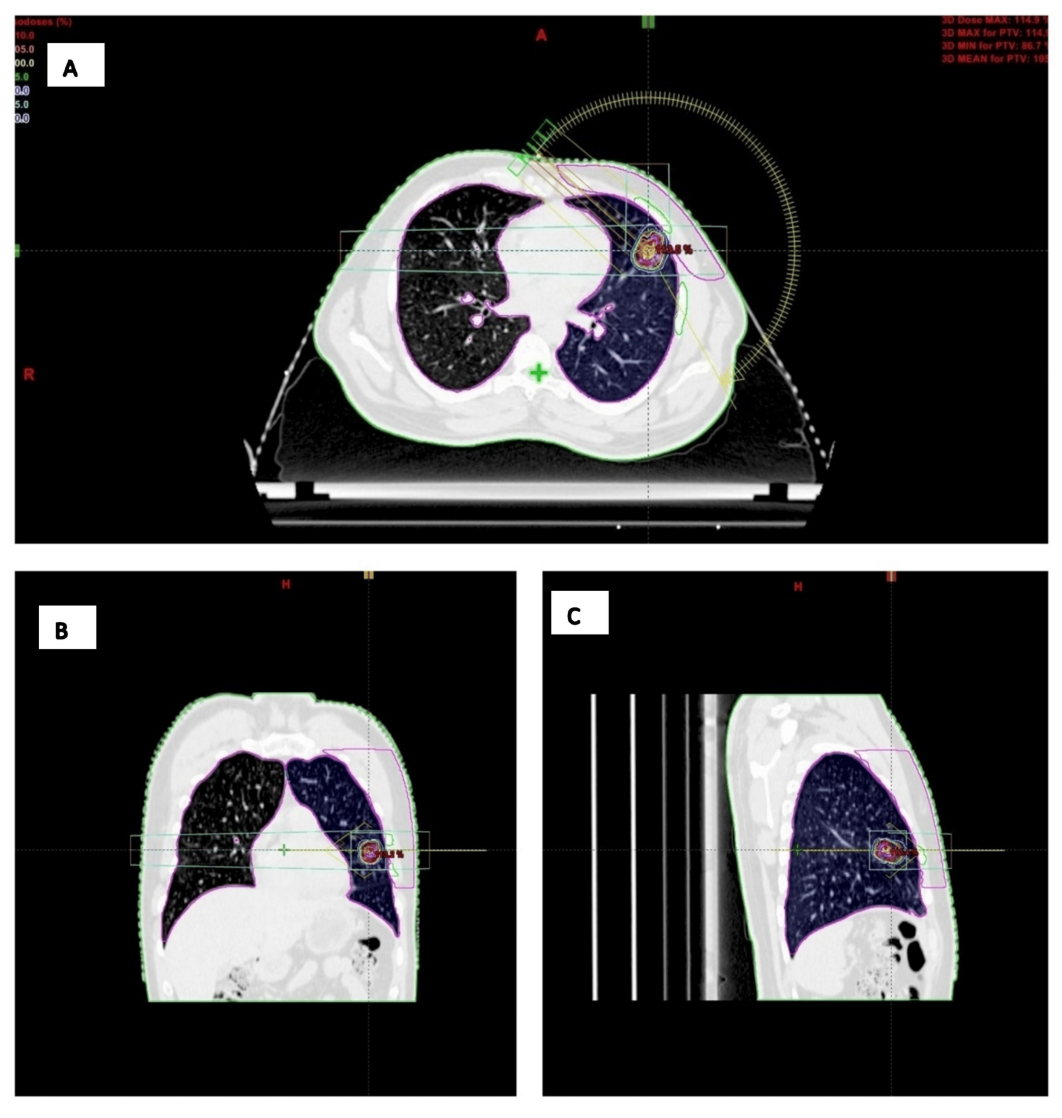
Thoracic CT-based radiotherapy treatment planning with delineation of the primary lung tumor during deep inspiratory breath-hold (DIBH). A: Axial (transverse) CT view of the thorax, showing a cross-section through the chest at the level of the lung lesion with the target volume, organs at risk (lungs, chest wall), and radiation dose distribution overlaid. (B) Coronal view, which slices the body from front to back and illustrates the superior-inferior extent of the target relative to both lungs and surrounding structures. (C) Sagittal view, a side-on slice of the thorax, highlighting the anterior-posterior relationship of the lesion and the beam geometry across the lung. A lung lesion near the chest wall is shown (surrounded by colored contours). Isodose lines (concentric colored rings) demonstrate radiation dose fall-off around the target; beam and arc geometry (yellow arcs and lines) illustrate the angles of radiation delivery; organs at risk are delineated in pink, most prominently the lung on the treated side and the heart; the external patient contour is outlined in green.

**Figure 2:**
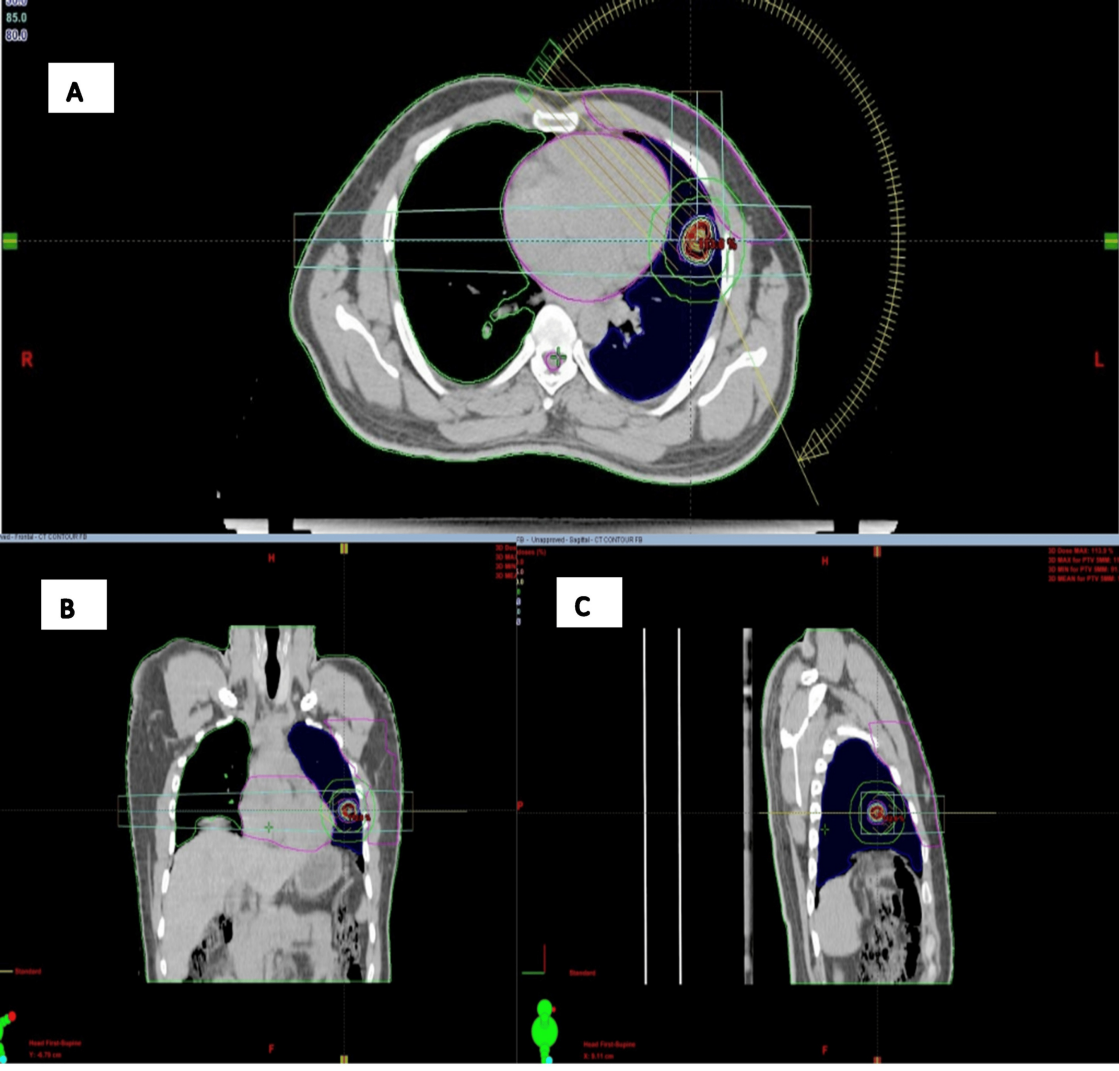
Thoracic CT-based radiotherapy treatment planning system with delineation of the primary lung tumor during free breathing. (A) Axial (transverse) CT slice through the chest, illustrating the lungs, heart, and a target lesion with superimposed target contours and isodose distributions. (B) Coronal view, providing a front-to-back perspective of the thorax that demonstrates the cranio-caudal extent of the target volume relative to surrounding organs at risk, particularly the lungs and mediastinum. (C) Sagittal view, offering a side profile of the thorax that highlights the anterior-posterior relationship between the target, lung tissue, and adjacent structures, along with the corresponding dose distribution. A lung lesion near the chest wall is shown (surrounded by colored contours). Isodose lines (concentric colored rings) demonstrate radiation dose fall-off around the target; beam and arc geometry (yellow arcs and lines) illustrate the angles of radiation delivery; organs at risk are delineated, most prominently the lung on the treated side (blue), the heart (pink), and the spinal cord (pink); the external patient contour is outlined in green.

Treatment planning was performed using the Eclipse Treatment Planning System (Varian Medical Systems, Palo Alto, CA) with the anisotropic analytical algorithm (AAA) for dose calculation. A 6 MV flattening filter-free (FFF) photon beam was used. Plans were generated using two coplanar partial arcs with a 180°-220° offset. A dose grid resolution of 2.5 mm was applied, and heterogeneity correction was enabled. Identical beam geometry, prescription dose, and OAR constraints were used for both FB and DIBH plans to ensure direct comparability. The prescribed SBRT dose ranged from 50 to 60 Gy delivered in 3-5 fractions, depending on tumor size and location, ensuring a BED10 ≥ 100 Gy. Dose was prescribed such that at least 95% of the PTV received 100% of the prescribed dose, while respecting established OAR constraints. Dosimetric evaluation was performed using dose-volume histograms (DVHs). Target coverage parameters included D2%, D50%, D98%, V95%, conformity index (CI), and homogeneity index (HI). Lung dose parameters analyzed included mean lung dose (MLD), V5, V10, V15, and V20. Doses to other OARs, such as the heart (mean dose), spinal cord (maximum dose), and chest wall (maximum dose), were recorded and compared between techniques (Figures 1, 2).

Statistical analysis

Statistical analysis was performed using the IBM SPSS Statistics for Windows, Version 20.0 (Released 2011; IBM Corp., Armonk, NY, USA). As both FB and DIBH plans were generated for the same patients, within-subject paired comparisons were conducted. Continuous variables were expressed as mean ± standard deviation. The paired Student’s t-test was used to compare dosimetric parameters between FB and DIBH plans, as the data represented normally distributed paired measurements within the same cohort. All tests were two-sided, and a p-value < 0.05 was considered statistically significant. Given that multiple dosimetric endpoints were evaluated, the analysis should be interpreted within an exploratory framework, as no formal adjustment for multiplicity was applied.

Ethical approval

The study was approved by the Institutional Ethics Committee of Yashoda Academy of Medical Education and Research (IEC-YAMER), Yashoda Hospital (approval number: DNBT-33/2018; dated March 21, 2018).

## Results

A total of 20 patients eligible for SBRT were included in the study, with a mean age of 52.5 years (range: 41-68 years). The cohort comprised 14 males and 6 females, with a smoking history present in 6 male patients. Tumor laterality showed a predominance of right-sided lesions (n = 13) compared to left-sided tumors (n = 7). Most tumors were peripherally located, and the majority of patients had associated comorbidities, such as chronic obstructive pulmonary disease (COPD), hypertension, diabetes mellitus, or cardiac disease. Histopathological subtypes included adenocarcinoma, squamous cell carcinoma, and large cell carcinoma. DIBH CBCT demonstrated superior tumor visualization and sharper target boundary definition compared to FB imaging, facilitating improved target delineation and contour confidence during planning (Table 1).

**Table 1:** Patient baseline demographics, tumor staging, location, comorbidities, and histology. COPD: chronic obstructive pulmonary disease, M: male, F: female.

S. no.	Stage	Age (in years)	Gender	Location	Comorbidities	Histology
1	T2aN0M0	62	M	Right lower	Hypertension	Adenocarcinoma
2	T2aN0M0	48	M	Left lower	COPD	Squamous
3	T2bN0M0	53	F	Left upper	-	Large cell
4	T2aN0M0	65	M	Right lower	Diabetes	Adenocarcinoma
5	T2aN0M0	49	M	Right upper	Heart disease	Adenocarcinoma
6	T1cN0M0	50	F	Right middle	Diabetes	Squamous
7	T1bN0M0	51	M	Right lower	COPD	Adenocarcinoma
8	T1bN0M0	67	M	Left lower	-	Squamous
9	T2aN0M0	49	M	Left upper	-	Adenocarcinoma
10	T2bN0M0	53	F	Right lower	Diabetes	Squamous
11	T1cN0M0	46	F	Right upper	Heart disease	Squamous
12	Stage IV	59	M	Right middle	Hypertension	Adenocarcinoma
13	T1bN0M0	68	M	Right lower	COPD	Large cell
14	Stage IV	67	F	Left lower	Diabetes	Adenocarcinoma
15	Oligometastatic	62	M	Left upper	Hypertension	Squamous
16	T1cN0M0	43	M	Right lower	-	Adenocarcinoma
17	T1bN0M0	58	F	Right upper	-	Squamous
18	T2aN0M0	55	M	Right middle	COPD	Adenocarcinoma
19	T2bN0M0	47	M	Left upper	Diabetes	Adenocarcinoma
20	T2aN0M0	41	M	Right lower	Hypertension	Squamous

Plan quality analysis demonstrated a significant improvement in the conformity index (CI) with the use of the DIBH technique compared to FB, irrespective of tumor site and lung volume. The mean CI (± SD) was 1.05 ± 0.08 for DIBH-SBRT and 1.02 ± 0.11 for FB-SBRT, indicating superior dose conformity with breath-hold planning. However, homogeneity indices (HI) were comparable between the two techniques, with no statistically significant difference observed. Dose distribution parameters including D2%, D50%, and D98% were also similar between the two approaches, confirming that improved conformity with DIBH did not compromise dose homogeneity within the PTV (Table 2).

**Table 2:** Comparison of conformity index and homogeneity index between DIBH SBRT and FB SBRT. *Statistically significant. Statistical comparison between DIBH and FB SBRT plans was performed using a paired Student’s t-test, as both techniques were applied to the same patient cohort. D2%: dose received by the hottest 2% of the planning target volume (representing near-maximum dose), D50%: median dose received by 50% of the PTV, D98%: dose received by 98% of the planning target volume (representing near-minimum dose), CI (RTOG): conformity index as per the Radiation Therapy Oncology Group definition, HI: homogeneity index, PTV: planning target volume, DIBH SBRT: deep inspiration breath-hold stereotactic body radiotherapy, FB SBRT: free-breathing stereotactic body radiotherapy.

Parameter	DIBH	FB	Test statistic (t-value)	p-value
	Mean ± SD			
D2% (Gy)	55.08 ± 10.34	53.56 ± 11.75	1.21	0.241
D50% (Gy)	52.46 ± 9.88	52.91 ± 10.16	-0.68	0.505
D98% (Gy)	49.11 ± 9.48	47.71 ± 10.08	1.34	0.195
95% isodose volume (cc)	127.33 ± 107.67	131.20 ± 106.45	-0.74	0.468
PTV volume (cc)	113.26 ± 110.70	123.41 ± 104.59	-1.89	0.073
CI (RTOG)	1.05 ± 0.08	1.02 ± 0.11	2.17	0.043*
HI	0.11 ± 0.02	0.11 ± 0.03	0.09	0.928

**Figure 3:**
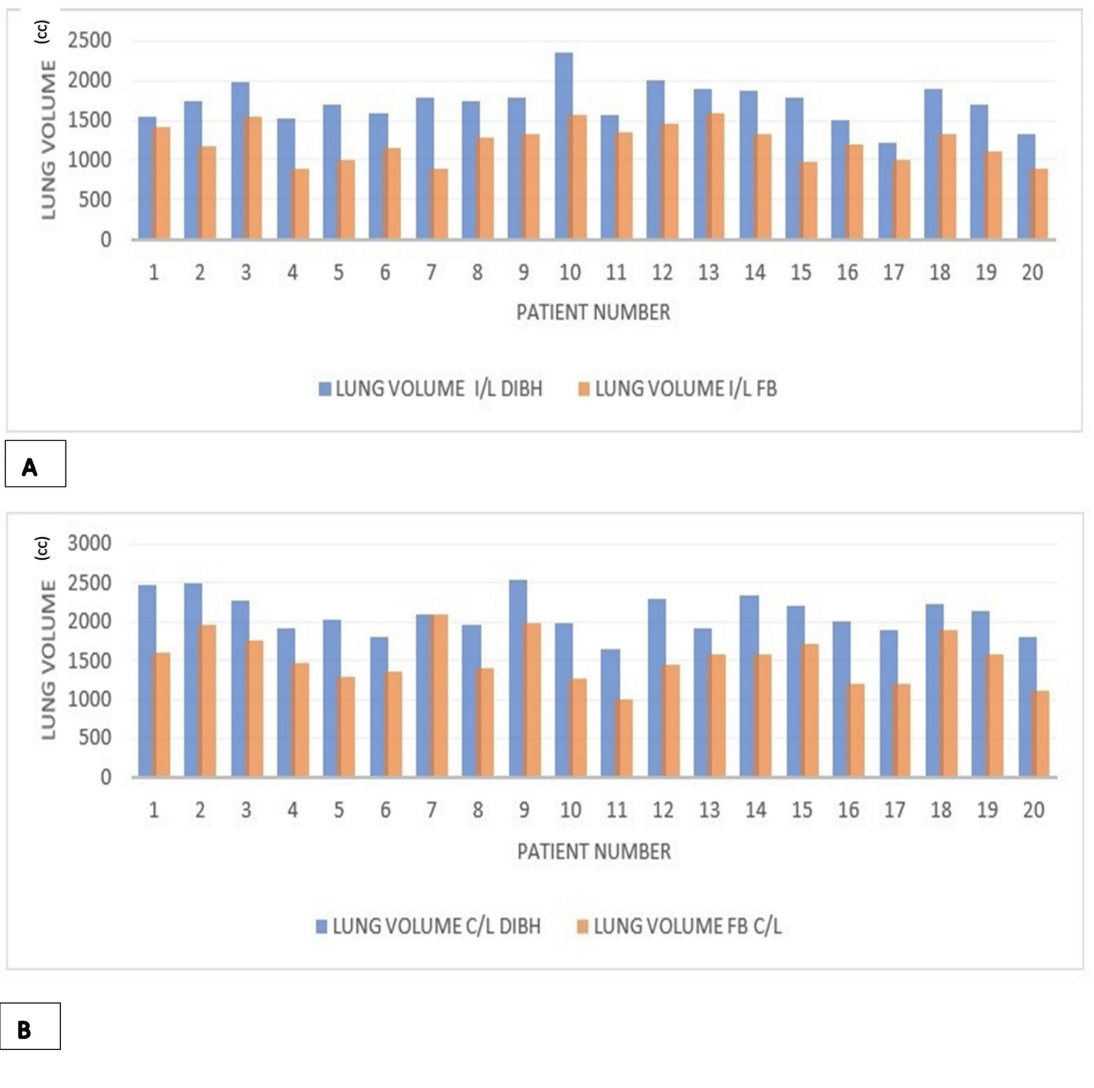
Lung volume comparison. (A) Ipsilateral (I/L) lung volume comparison for deep inspiratory breath hold (DIBH) and free-breathing (FB) maneuvers. (B) Contralateral (C/L) lung volume comparison for DIBH and FB maneuvers. Blue: DIBH volumes. Orange: FB volumes.

Automatic lung contouring revealed a significant increase in total lung volume during DIBH acquisitions compared to FB, with no observed gender-based differences. Mean ipsilateral and contralateral lung volumes increased by factors of 1.41 and 1.37, respectively, during DIBH. This volumetric expansion translated into significant reductions across all evaluated lung dosimetric parameters (Figure 3).

For the ipsilateral lung, statistically significant reductions were observed in V10, V15, V20, and MLD (Dmean), with relative reductions of 33.51%, 40.49%, 51.95%, and 37.34%, respectively (all p < 0.001). Similarly, the contralateral lung showed a significant reduction in mean dose, with Dmean decreasing from 11.75 ± 3.90 Gy in FB to 8.56 ± 3.39 Gy in DIBH plans (17.56% reduction; p = 0.0085). Overall, DIBH SBRT resulted in a MLD reduction of approximately 37% compared to FB-SBRT. Significant dosimetric advantages of DIBH were also observed for non-pulmonary critical structures. The mean heart dose was significantly reduced in DIBH plans compared to FB plans (p = 0.0027), with a relative dose reduction of 8.56%. Similarly, the maximum dose (Dmax) to the spinal cord was significantly lower with DIBH SBRT (26.46 ± 8.59 Gy) compared to FB-SBRT (28.73 ± 7.98 Gy), representing an 8.58% reduction (p = 0.0027). Chest wall doses followed a similar trend, with DIBH plans demonstrating a statistically significant reduction in maximum dose (34.88 ± 11.35 Gy vs 38.44 ± 11.77 Gy), corresponding to a 10.2% reduction (p = 0.004). These findings indicate consistent sparing of adjacent critical organs with the DIBH technique (Table 3).

**Table 3:** Comparison of lung dosimetric parameters and organ-at-risk (OAR) doses between DIBH and FB techniques. Statistical comparison between deep inspiration breath-hold (DIBH) and free-breathing (FB) SBRT plans was performed using a paired Student’s t-test, as both techniques were evaluated in the same patient cohort. V10, V15, V20: percentage volume of lung receiving ≥10 Gy, ≥15 Gy, and ≥20 Gy, respectively.

Parameter	DIBH (mean ± SD)	FB (mean ± SD)	Difference (%)	Test statistic (t-value)	p-value
	Mean ± SD				
Ipsilateral lung					
Volume (cc)	1724 ± 254.04	1221 ± 227.03	-	8.92	<0.0001
V10 (%)	24.12 ± 13.26	32.27 ± 14.12	33.51	-6.87	<0.0001
V15 (%)	18.62 ± 10.75	26.16 ± 12.92	40.49	-7.43	<0.0001
V20 (%)	12.35 ± 9.10	18.76 ± 11.12	51.95	-8.15	<0.0001
Mean dose (Gy)	8.56 ± 3.39	11.75 ± 3.90	37.34	-9.02	<0.0001
Contralateral lung					
Volume (cc)	2103 ± 251.29	1524.78 ± 308.15	-	7.96	<0.0001
Mean dose (Gy)	2.38 ± 0.92	2.79 ± 1.10	17.56	-2.98	0.0009
Heart					
Mean dose (Gy)	11.19 ± 5.45	13.32 ± 6.73	19	-3.91	0.0002
Spinal cord					
Maximum dose (Gy)	26.46 ± 8.59	28.73 ± 7.98	8.58	-3.47	0.0027
Chest wall					
Maximum dose (Gy)	34.88 ± 11.35	38.44 ± 11.71	10.2	-4.21	0.0004

Target coverage analysis revealed no statistically significant differences between FB and DIBH techniques. Coverage parameters V95%, V97%, and V99% were comparable across both planning approaches. Mean V95% values were 97.83 ± 1.06% for FB and 98.25 ± 0.97% for DIBH (p = 0.197), while V97% and V99% also showed no significant differences (p = 0.176 and p = 0.376, respectively). Similarly, plan quality indices remained consistent, with CI and HI values demonstrating no clinically meaningful variation between the two techniques. These results confirm that the observed dosimetric benefits of DIBH were achieved without compromising target coverage or plan quality (Table 4). 

**Table 4:** Comparison of the target coverage parameters between FB SBRT and DIBH SBRT. Statistical comparison between free-breathing (FB) and deep inspiration breath-hold (DIBH) stereotactic body radiotherapy (SBRT) plans was performed using a paired Student’s t-test, as both planning techniques were applied to the same patient cohort. The p-values and corresponding t-statistics represent comparisons of the mean target coverage values between FB and DIBH plans. V95, V97, V99: percentage of planning target volume (PTV) receiving at least 95%, 97%, and 99% of the prescribed dose, respectively.

Parameter	FB min (%)	FB max (%)	FB (mean ± SD, %)	DIBH min (%)	DIBH max (%)	DIBH (mean ± SD, %)	Test statistic (t-value)	p-value
V95	96.1	99.62	97.83 ± 1.06	96.3	99.68	98.25 ± 0.97	-1.34	0.197
V97	78.6	97.39	90.80 ± 5.05	89.32	98.63	93.97 ± 4.21	-1.42	0.176
V99	68.23	93.21	82.29 ± 7.72	71.58	95.65	85.38 ± 7.38	-0.90	0.376

## Discussion

Radiotherapy for lung cancer is inherently challenged by respiratory-induced tumor motion, which remains one of the principal contributors to geometric uncertainty and inadvertent irradiation of surrounding healthy lung tissue [16]. This motion-related uncertainty is particularly critical in SBRT, where high doses per fraction and steep dose gradients mandate precise target localization and motion control [17]. The present study was undertaken to evaluate whether DIBH, as an advanced motion management strategy, offers dosimetric advantages over conventional FB SBRT in patients with localized lung tumors treated at a tertiary care center.

A principal finding of this study was the significant increase in lung volume observed during DIBH compared with FB imaging. Lung inflation during deep inspiration leads to expansion of aerated lung tissue and increased spatial separation between normal lung parenchyma and the target volume, thereby reducing the relative lung volume exposed to radiation [18]. In our cohort, ipsilateral and contralateral lung volumes increased by factors of 1.41 and 1.37, respectively. These findings are consistent with previously published data; Lansing et al. and Wang et al. reported mean lung volume increases of approximately 57% with DIBH compared to FB in comparative planning studies, supporting the reproducibility of this physiological effect across different patient populations and clinical settings [19,20].

The volumetric expansion translated into statistically significant reductions in low- and intermediate-dose lung parameters, including V10, V15, V20, and MLD. These parameters are clinically relevant, as radiation pneumonitis has been strongly associated with lung dose-volume metrics, particularly V20 and MLD. Symptomatic radiation pneumonitis has been reported in nearly 30% of patients following thoracic radiotherapy, with V20 widely regarded as a surrogate marker for pulmonary toxicity, as also observed in studies by Harder et al., Moiseenko et al., and Ryckman et al. [21-23]. In our study, DIBH SBRT resulted in a 51.95% reduction in ipsilateral lung V20 and a 37.34% reduction in MLD compared to FB SBRT (p < 0.001). While this represents a substantial dosimetric improvement, it must be emphasized that clinical toxicity outcomes were not assessed in the present study; therefore, the reduction in pneumonitis risk remains inferential.

Studies by Ricardi et al. and Ji et al. have suggested that maintaining the pulmonary mean dose below 15-20 Gy may reduce the risk of long-term lung toxicity [24,25]. Importantly, all DIBH plans in our study satisfied these recommended constraints. The observed reduction in MLD from 11.75 ± 3.90 Gy in FB plans to 8.56 ± 3.39 Gy in DIBH plans supports the lung-sparing potential of DIBH within established safety thresholds [24,25]. However, as this study was dosimetric in nature, the implications for long-term fibrosis or functional outcomes warrant prospective validation.

Cardiac dose reduction was another significant finding. Although cardiac toxicity is more commonly discussed in conventionally fractionated thoracic radiotherapy, emerging evidence indicates that even moderate cardiac radiation exposure may contribute to long-term morbidity through mechanisms such as microvascular injury, accelerated atherosclerosis, and inflammatory remodeling [26]. Epidemiological data in thoracic radiotherapy suggest a dose-dependent increase in late cardiac events, even at intermediate dose ranges [26]. In the present study, the mean heart dose was significantly reduced in DIBH plans compared to FB plans (p = 0.0027), with an approximate reduction of 8.56%. Although advanced predictive parameters such as V40 and D100, previously associated with cardiac toxicity at cut-off values of around 30% and 30 Gy, respectively, were not evaluated, the observed reduction in mean heart dose suggests a potential reduction in cumulative cardiac exposure [26]. Nevertheless, clinical cardiotoxicity endpoints were not assessed, and therefore clinical benefit should not be presumed.

Similarly, doses to other critical OARs demonstrated statistically significant reductions with DIBH. The maximum dose to the spinal cord was reduced by 8.58% (p = 0.0027), and the maximum dose to the chest wall decreased by 10.2% (p = 0.004). These reductions likely reflect reduced tumor motion, improved geometric precision, and favorable anatomical displacement during deep inspiration [27]. Lower chest wall doses are particularly relevant in peripheral lung SBRT, as chest wall pain and rib fractures represent recognized late toxicities [27].

Importantly, these dosimetric advantages were achieved without compromising target coverage or plan quality. Coverage parameters (V95%, V97%, V99%), CI, and HI were comparable between DIBH and FB plans. This finding aligns with SBRT planning studies by Kawahara et al. and Liu et al., demonstrating that motion mitigation strategies can improve normal tissue sparing while maintaining adequate target coverage [28,29]. While the concept of an improved therapeutic ratio is supported dosimetrically, it remains an extrapolation pending outcome-based validation.

Most tumors in this cohort were peripherally located, which may explain the relatively low baseline doses to centrally located structures such as the esophagus, pulmonary trunk, and carina. Consequently, these organs were not analyzed in detail. The feasibility of DIBH SBRT is also dependent on patient cooperation and breath-hold reproducibility [30]. Structured patient training and gated DIBH CBCT were used in this study to enhance delivery accuracy, and the majority of patients demonstrated satisfactory compliance [30]. However, this approach may not be suitable for patients with severe pulmonary compromise.

Limitations

Several limitations should be acknowledged. First, the sample size (n = 20) was relatively small, which may limit generalizability and statistical power. Second, this was a dosimetric planning study without clinical outcome assessment; therefore, reductions in lung and heart doses cannot be directly equated with reduced pneumonitis, cardiotoxicity, or improved survival. Third, most tumors were peripherally located, limiting evaluation of centrally located OAR dosimetry. Fourth, DIBH feasibility is inherently dependent on patient cooperation and breath-hold reproducibility. Fifth, advanced cardiac dose-volume parameters, such as V40 and D100, were not analyzed and should be incorporated in future studies. Finally, multiple dosimetric comparisons were performed without formal adjustment for multiplicity, which may increase the risk of type I error. Accordingly, the findings should be interpreted within the exploratory dosimetric context of this study.

## Conclusions

This paired dosimetric comparison demonstrates that DIBH SBRT provides measurable dosimetric advantages over conventional FB SBRT for localized lung tumors. DIBH SBRT was associated with significant reductions in lung dose parameters (V10, V15, V20, and MLD) as well as decreased radiation exposure to other critical OARs, including the heart, spinal cord, and chest wall, without compromising target coverage, conformity, or homogeneity. The observed benefits are likely attributable to lung volume expansion and reduced respiratory-induced target motion during deep inspiration. These findings support the role of DIBH as an effective motion management strategy in lung SBRT from a dosimetric perspective. However, as this study was limited to treatment planning analysis, the observed improvements in dose distribution should not be directly interpreted as proven reductions in clinical toxicity or improved survival outcomes. The potential for enhancement of the therapeutic balance remains inferential and warrants validation through larger prospective studies incorporating long-term toxicity and clinical outcome data. With appropriate patient selection, structured breath-hold training, and image-guided verification, DIBH may be considered a valuable adjunct in selected patients undergoing lung SBRT.
